# Deep-Neural-Network-Based Modelling of Longitudinal-Lateral Dynamics to Predict the Vehicle States for Autonomous Driving

**DOI:** 10.3390/s22052013

**Published:** 2022-03-04

**Authors:** Xiaobo Nie, Chuan Min, Yongjun Pan, Ke Li, Zhixiong Li

**Affiliations:** 1 College of Mechanical and Vehicle Engineering, Chongqing University, Chongqing 400044, China; xiaobo.nie@cqu.edu.cn (X.N.); chuan.min@cqu.edu.cn (C.M.); 2 State Key Laboratory of Structural Analysis for Industrial Equipment, Dalian University of Technology, Dalian 116024, China; 3 School of Civil Engineering, Chongqing University, Chongqing 400044, China; keli-bridge@cqu.edu.cn; 4 Faculty of Mechanical Engineering, Opole University of Technology, 45758 Opole, Poland; z.li@po.edu.pl

**Keywords:** deep neural networks, longitudinal-lateral dynamics, autonomous vehicle, real-time simulation

## Abstract

Multibody models built in commercial software packages, e.g., ADAMS, can be used for accurate vehicle dynamics, but computational efficiency and numerical stability are very challenging in complex driving environments. These issues can be addressed by using data-driven models, owing to their robust generalization and computational speed. In this study, we develop a deep neural network (DNN) based model to predict longitudinal-lateral dynamics of an autonomous vehicle. Dynamic simulations of the autonomous vehicle are performed based on a semirecursive multibody method for data acquisition. The data are used to train and test the DNN model. The DNN inputs include the torque applied on wheels and the vehicle’s initial speed that imitates a double lane change maneuver. The DNN outputs include the longitudinal driving distance, the lateral driving distance, the final longitudinal velocities, the final lateral velocities, and the yaw angle. The predicted vehicle states based on the DNN model are compared with the multibody model results. The accuracy of the DNN model is investigated in detail in terms of error functions. The DNN model is verified within the framework of a commercial software package CarSim. The results demonstrate that the DNN model predicts accurate vehicle states in real time. It can be used for real-time simulation and preview control in autonomous vehicles for enhanced transportation safety.

## 1. Introduction

Recent advances on neural networks have led to dramatic progress in solving many complex engineering problems, and demand for such solutions is increasing with time. A wide range of topics starting from dynamics modelling, intelligent control to transportation research is being handled by neural networks [1,2,3]. This technique has naturally found substantial applications in autonomous vehicles, with vehicle lateral and longitudinal dynamics modelling and control being typical applications [4,5,6]. The advent of autonomous vehicles has the potential to drastically change society and the way we understand, plan and design cities and regions [7,8,9]. It will make transportation smoother, but the question is whether it will make it safer [10,11]. For preview control of autonomous vehicles in terms of accuracy and safety, it is critical to investigate the coupling between lateral and longitudinal dynamics for accurate data acquisition, which is necessary for deep learning modelling [12,13,14]. This process requires highly detailed vehicle models or large sets of raw data to properly take into account.

In recent years, a number of contributions have been reported in the field of vehicle coupling dynamics modelling and control by using neural networks. Kumarawadu et al. proposed a neural network adaptive control approach for longitudinal-lateral dynamics of vehicles for highway applications. The results in the face of parameter uncertainties indicated the stability and robustness of the control algorithm [15]. Melzi et al. presented a layered neural network method that uses dynamic parameters acquired from sensors to estimate the sideslip angle. The results showed good consistency between the measured and estimated sideslip angles [16]. Ji et al. developed a lateral motion control strategy by using an adaptive neural network approximator and a robust steering controller. The results suggested that the strategy can track the path and maintain yaw stability at the physical limits of tire friction [17]. Devineau et al. analyzed the capability of deep neural networks (DNNs) to capture vehicle characteristics and the ability to perform longitudinal-lateral control [18]. Acosta et al. integrated feedforward neural networks into a model predictive controller to achieve vehicle autonomous drifting along a wide range of road radii [19]. Taghavifar et al. developed a probabilistic estimation method by hybridization of least-square backpropagation neural networks and optimal robust control. The approach improved vehicle handling and stability [20]. Kim et al. introduced a sideslip angle estimation scheme by combining DNNs and nonlinear Kalman filters. The scheme was verified by both simulation and experimental results [21]. Tork et al. performed longitudinal-lateral dynamics control in an autonomous vehicle system. An adaptive neural network that is capable of producing nonlinear and complex mappings was designed [22]. Šabanovič developed a road type classification solution to improve vehicle dynamics control via the anti-lock braking system by estimating friction coefficient using video data and DNN algorithms [23].

The literature survey indicates that DNNs can be efficiently applied in vehicle dynamics modelling and control [24]. In most cases, longitudinal-lateral modelling problems were investigated in a decoupled way. The precise modelling of longitudinal-lateral coupling involves complex and nonlinear relations between vehicle state variables, and using the resulting vehicle model is too costly for real-time applications. For this reason, most studies in the field of preview control mainly focus on simplified or few-degree-of-freedom models, which are constrained to avoid highly coupled dynamics [25,26]. However, the simultaneous inclusion of longitudinal and lateral control becomes unavoidable to improve vehicle performance in a wide range of operations especially for autonomous vehicles [27,28]. The major difficulty of DNN-based methods for longitudinal-lateral dynamics is the number of driving situations required for building a representative training dataset. The direct estimation of vehicle characteristics is sometimes unavailable since numerous road tests of vehicles are needed. Vehicle dynamic simulations within the framework of commercial software packages are also time-consuming because real-time simulations are very challenging in low-cost hardware [29,30].

To address these issues, a data-driven modelling method based on real-time data acquisition and DNNs provides an efficient solution. In this study, a DNN model for predicting longitudinal-lateral dynamics of a vehicle is presented. An efficient semirecursive multibody method that performs real-time simulations is used to capture vehicle key characteristics. The highlights of this study lies in two aspects. The first aspect consists of the use of an efficient semirecursive multibody formulation to acquire training and testing data. The multibody model can deal with the nonlinearities of vehicle systems more accurately than the simplified or decoupled models. The second aspect consists of a DNN modelling approach to predict longitudinal-lateral dynamics of a vehicle. Different applied torques and initial speeds over a wide range are used to imitate various kinds of on-road driving situations. The DNN model is verified by using the results obtained from a commercial software package CarSim. Further, the DNN models with different hidden layers and sample size are investigated in terms of accuracy and efficiency. The widely used backpropagation neural network and radical basis function neural network are employed as references to tell the advantages of the DNN model used to predict the vehicle states.

The rest of the study is organized as follows. In Section 2, an efficient semirecursive multibody method is introduced to develop a vehicle model and collect the vehicle data for DNN modelling. A DNN model with multiple inputs and outputs for predicting the lateral-longitudinal dynamics of an autonomous vehicle is developed. In Section 3, the results of the DNN model are comprehensively investigated in accuracy and efficiency, and the effectiveness of the DNN model is verified within the framework of a commercial software CarSim. Finally, in Section 4, we conclude our work.

## 2. DNN-Based Modelling Methodology for Vehicle Dynamics

### 2.1. Vehicle Multibody Method for Data Acquisition

In this section, we used an efficient semirecursive multibody method to develop a vehicle dynamics model. The equations of motion of the closed-loop vehicle systems can be formulated in terms of independent relative (joint) coordinates. They can be expressed as [31,32]:

(1) $$R_{z}^{T}R_{d}^{T}M^{\Sigma }R_{d}R_{z}{\ddot{z}}^{i}=R_{z}^{T}R_{d}^{T}\left[Q^{\Sigma }-T^{T}\bar{M}\frac{d\left(TR_{d}R_{z}\right)}{dt}{\dot{z}}^{i}\right]$$

where, $R_{d}$ and $R_{z}$ denote the first and second velocity transformation matrices, which describe the Cartesian velocities and accelerations by means of independent relative velocities and accelerations. The superscript T denotes the transpose operation. $\bar{M}$ denotes the composite mass matrix of the multibody system. $M^{\Sigma }$ and $Q^{\Sigma }$ contain the accumulated generalized mass matrix and external forces, respectively. T denotes the path matrix, representing the system recursive connectivity. ${\dot{z}}^{i}$ and ${\ddot{z}}^{i}$ contain the independent relative (joint) velocities and accelerations, respectively.

This formulation is called the double-step semirecursive multibody formulation, which was proposed by Javier García de Jalón and his coworkers [33,34]. Although Equation (1) is more complicated than other multibody formulations, it uses a small set of independent relative accelerations ${\ddot{z}}^{i}$, which leads to higher computational efficiency. The ordinary differential form of the equations of motion enables the stable simulation of vehicle systems via various numerical integrators [35,36]. Low-order integrators are very suitable for efficient vehicle simulations. However, the solution accuracy is unable to satisfy the requirement as the numerical error accumulates over time. The Adams–Bashforth–Moulton integrator and the 4th-order Runge–Kutta integrator are typical high-order integrators that are often used in real-time simulations with longer simulation times.

The training and testing sets of vehicle dynamics data can be obtained in real time based on the semirecursive multibody formulation. Thus, we aim to collect historical data that include the torque applied on wheels, the vehicle’s initial speed, the longitudinal driving distance, the lateral driving distance, the final longitudinal velocities, the final lateral velocities, and the yaw angle to build a lateral-longitudinal dynamics model by using a DNN approach. The capacity of the DNN modelling approach to capture the key vehicle states is explored, and verified based on the results obtained from a commercial software package CarSim. The data-driven vehicle model is developed offline via DNN approach and used online for preview control in autonomous vehicles. The computational burden of the data-driven model is much smaller than that of the vehicle multibody models, e.g., ADAMS model, CarSim model, and semirecursive multibody model. Furthermore, the data-driven model avoids the numerical integration process of vehicle multibody models, and it leads to robust generation of the vehicle states.

### 2.2. DNN Structure of the Vehicle Dynamics

**Figure 1:**
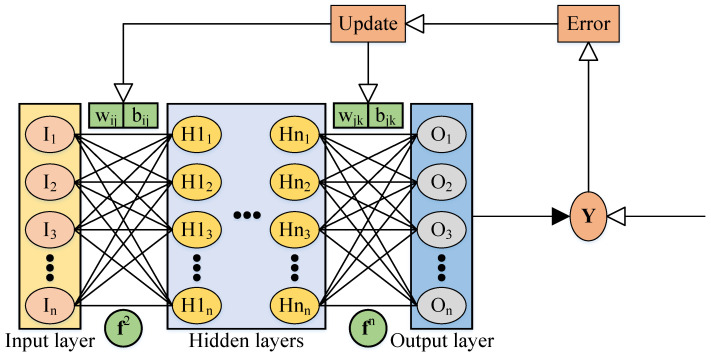
Deep neural network structure.

DNNs can learn and store relationships between the input and output neurons. Mathematical theory has proved that DNNs can approach any nonlinear continuous functions with high precision. A general DNN framework is described in Figure 1.

The DNN training process is composed of forward propagation of inputs and backpropagation of errors. The input matrix, the weight matrices, and the bias matrices in forward propagation are described as:

(2) $$\begin{array}{cc} \; & Z_{1}\;=\;\left(i_{1},\;i_{2},\;i_{3},\;\cdots \;i_{m}\right) \end{array}$$

(3) $$\begin{array}{cc} \; & W_{n}=\left(w_{n_{1}},\;w_{n_{2}},\;w_{n_{3}},\;\cdots \;w_{n_{m}}\right) \end{array}$$

(4) $$\begin{array}{cc} \; & B_{n}=\left(b_{n_{1}},\;b_{n_{2}},\;b_{n_{3}},\;\cdots \;b_{n_{m}}\right) \end{array}$$

where, $Z_{1}$ denotes the input matrix, $W_{n}$ and $B_{n}$ denote the weight matrix and bias matrix of the *n*-th layer, respectively. *m* denotes the sample size in the training set, and *n* denotes the number of hidden layers and input layer,

The forward propagation steps are expressed as:

(5) $$\begin{array}{cc} \; & Z_{1}\;=\;A_{1} \end{array}$$

(6) $$\begin{array}{cc} \; & Z_{i+1}\;=\;W_{i}^{T}A_{i}+B_{i},\;i=1\ldots n \end{array}$$

(7) $$\begin{array}{cc} \; & A_{i+1}\;=\;f^{i+1}\left(Z_{i+1}\right),\;i=1\ldots n \end{array}$$

where, $Z_{i}$ and $A_{i}$ denote the input and output of the *i*-th layer, respectively, and $f^{i}$ denotes the *i*-th activation function. Note that $A_{n+1}$ contains the results of the DNN model. There are various types of activation functions to help the DNN model fit the linear and nonlinear functions.

The mean square error (MSE) was selected as the loss function to measure the differences between the DNN results and sample results before backpropagation. By implementing backpropagation, the weight matrices and bias matrices were updated to minimize the loss function. In this study, L2 regularization was used to calculate the loss function to avoid overfitting. The MSE, the L2 regularization, and the loss function are expressed as [37]:

(8) $$\begin{array}{cc} \; & \mathrm{MSE}=\frac{{∥Y-A_{n+1}∥}_{2}^{2}}{2m} \end{array}$$

(9) $$\begin{array}{cc} \; & L2=\frac{\lambda \Sigma {∥W_{n}∥}_{2}^{2}}{2m} \end{array}$$

(10) $$\begin{array}{cc} \; & e=\mathrm{MSE}+L2=\frac{{∥Y-A_{n+1}∥}_{2}^{2}+\lambda \Sigma {∥W_{n}∥}_{2}^{2}}{2m} \end{array}$$

where, *e* denotes the network’s cost, Y denotes the value of samples, and λ denotes the regularization coefficient.

The adaptive moment estimation optimization (Adam) algorithm was used in the backpropagation process. It can be regarded as a combination of the root mean square prop (RMSProp) algorithm and adaptive gradient (AdaGrad) algorithm [38]. The main parameters of AdaGrad and RMSProp are expressed as:

(11) $$\begin{array}{cc} \; & v_{dwn}=\beta_{1}v_{dwn}+\left(1-\beta_{1}\right)dW_{n} \end{array}$$

(12) $$\begin{array}{cc} \; & v_{dbn}=\beta_{1}v_{dbn}+\left(1-\beta_{1}\right)dB_{n} \end{array}$$

(13) $$\begin{array}{cc} \; & s_{dwn}=\beta_{2}s_{dwn}+\left(1-\beta_{2}\right){dW_{n}}^{2} \end{array}$$

(14) $$\begin{array}{cc} \; & s_{dbn}=\beta_{2}v_{dbn}+\left(1-\beta_{2}\right){dB_{n}}^{2} \end{array}$$

where, $dW_{n}$ and $dB_{n}$, respectively, denote the partial differential of the weight matrices and the bias matrices with respect to the loss function. $v_{dwn}$, $v_{dbn}$, $s_{dwn}$, and $s_{dbn}$ denote the AdaGrad and RMSProp parameters in each training iteration for weight matrices and bias matrices, respectively.

Because the moving exponential weighted average causes relatively large errors to the initial value at the beginning of the iteration, the AdaGrad and RMSProp parameters were updated by using the following expressions:

(15) $$\begin{array}{cc} \; & v_{dwn}^{c}=\frac{v_{dwn}}{1-\beta_{1}^{t}} \end{array}$$

(16) $$\begin{array}{cc} \; & v_{dbn}^{c}=\frac{v_{bdn}}{1-\beta_{1}^{t}} \end{array}$$

(17) $$\begin{array}{cc} \; & s_{dwn}^{c}=\frac{s_{dwn}}{1-\beta_{2}^{t}} \end{array}$$

(18) $$\begin{array}{cc} \; & s_{dbn}^{c}=\frac{s_{dbn}}{1-\beta_{2}^{t}} \end{array}$$

These parameters were corrected in each training iteration. The weight matrices and bias matrices, in turn, were updated based on the Adam algorithm:

(19) $$\begin{array}{cc} \; & W_{n}=W_{n}-\alpha \frac{v_{dwn}^{c}}{\sqrt{s_{dwn}^{c}}+\varepsilon } \end{array}$$

(20) $$\begin{array}{cc} \; & B_{n}=B_{n}-\alpha \frac{v_{dbn}^{c}}{\sqrt{s_{dbn}^{c}}+\varepsilon } \end{array}$$

where, ε denotes a smoothing parameter. It equals 10${}^{-8}$ in this vehicle example. α denotes the learning rate. It needs to be minutely updated in the DNN training process.

### 2.3. DNN Vehicle Model

**Figure 2:**
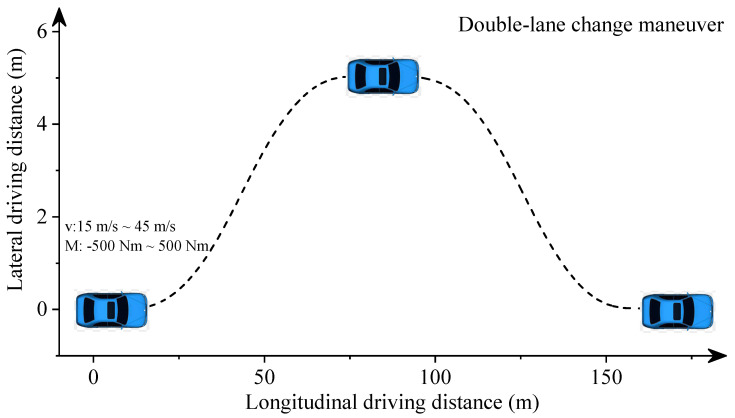
The driving track of the vehicle.

In this section, we used the semirecursive multibody formulation described by Equation (1) to obtain longitudinal-lateral dynamics datasets. The vehicle system consists of McPherson suspensions in the front axles, multilink suspensions in the rear axles, and Pacejka tire models [36,39]. In dynamic simulations, vehicles move with different initial speeds and front-wheel driving torques to imitate acceleration and deceleration situations. The simulations last for 5 s. The steering angle is controlled to ensure the double-lane change maneuver, as described in Figure 2.

We obtained critical vehicle states by introducing 500 different initial speeds and driving torques at defined steering angles. Those vehicle data, including the longitudinal distance and speed, the lateral distance and speed, and the yaw angle, denote the longitudinal-lateral dynamics of the autonomous vehicle. The range of the initial speed is 15 m/s to 45 m/s. The range of applied torque is −500 Nm to 500 Nm.

We randomly selected 500 samples and divided them into a training set with 450 samples and a testing set with 50 samples. The training and testing sets were used to train and test the DNN model. Furthermore, the training data were processed to eliminate the inconvenience caused by the magnitude of values. It can avoid small weight matrices, which may cause numerical instability during the integration. We used Z–score standardization to process the data, and the equation is described as:

(21) $$x^{*}=\frac{x-\mu }{\sigma }$$

where, $x^{*}$ represents the standardization value, *x* represents the value of the samples, μ represents the mean of the samples, and σ represents the standard deviation of the samples. The mean value and standard deviation of the processed data are 0 and 1, respectively.

There are two widely used methods to update the optimization parameters in DNNs. The first is batch gradient descent method that calculates all samples [40]. Its computational efficiency is low. The second is stochastic gradient descent method that computes the loss function of each sample and finds the updated parameters [41]. Its computational efficiency is high, but the convergence performance needs improvement. To overcome the drawbacks of these two methods, the minibatch gradient descent method was used in this work. This method divides the data into batches and updates the parameters in each batch. The data in a batch determines the direction of this gradient. It will not vanish easily when falling and will reduce the randomness. The batch size is set to 32 in this vehicle example.

**Figure 3:**
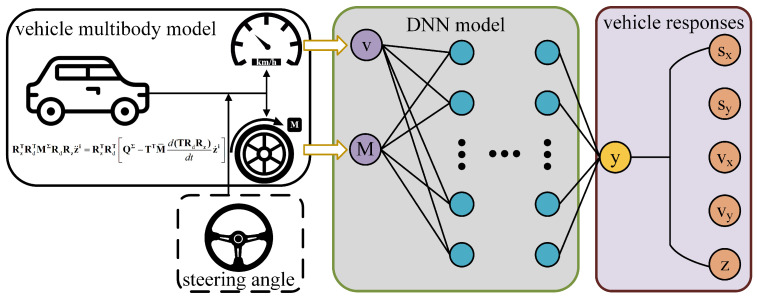
DNN model for vehicle dynamics.

By training the datasets, we built a DNN model to predict longitudinal-lateral dynamics of the autonomous vehicle. The DNN model for vehicle longitudinal-lateral dynamics is described in Figure 3.

The inputs of the DNN model include the initial speed and applied torque of the vehicle, as described by *v* and *M* in Figure 3. The outputs include the longitudinal distance and speed, the lateral distance and speed, and the yaw angle. They are described by $s_{x}$, $v_{x}$, $s_{y}$, $v_{y}$, and *z* in Figure 3, respectively. The DNN model involves 4 hidden layers, and each layer has 28, 24, 20, and 15 neurons. The activation functions are rectified linear unit (ReLU) functions, which are expressed as:

(22) $$f\left(x\right)=max\left(0,\;x\right)$$

**Figure 4:**
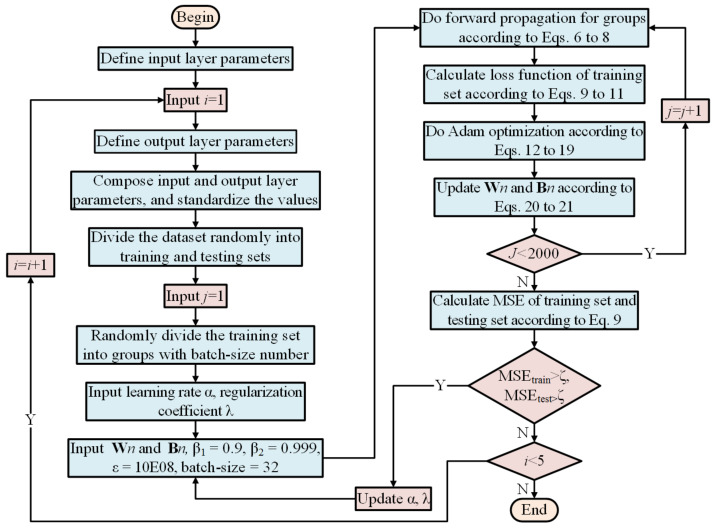
The procedure of DNN modelling.

When the DNN training is completed, the testing set is used for model test and verification. The MSE (loss function) of the dataset needs to be calculated. The DNN model can be used for the prediction and preview control if the MSE is very small. The procedure of DNN modelling for vehicle’s lateral-longitudinal dynamics is described in Figure 4.

## 3. DNN Model Results and Discussion

### 3.1. DNN Model Results

**Figure 5:**
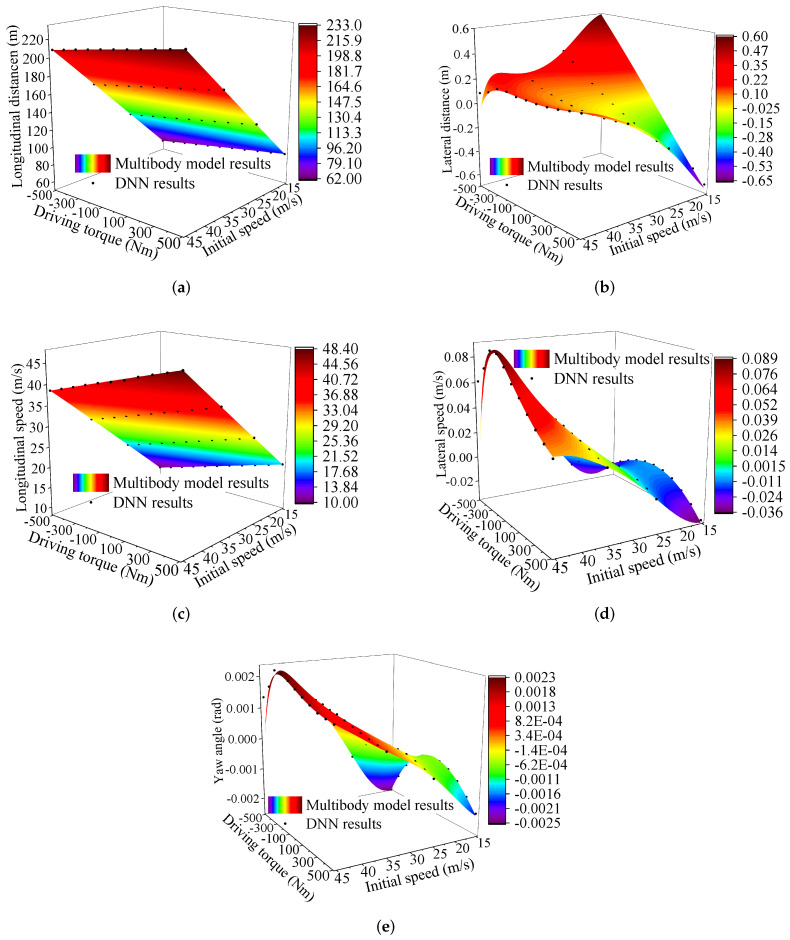
The comparative results. (**a**) Final longitudinal distance; (**b**) Final lateral distance; (**c**) Final longitudinal velocities; (**d**) Final lateral velocities; (**e**) Yaw angle.

In this section, the DNN model of the vehicle was developed. The DNN results were described and compared with the results of the multibody model. Figure 5 describes the result differences between the DNN model and the multibody model. Note that only part of the DNN results is shown in the figures for better visualization.

**Figure 6:**
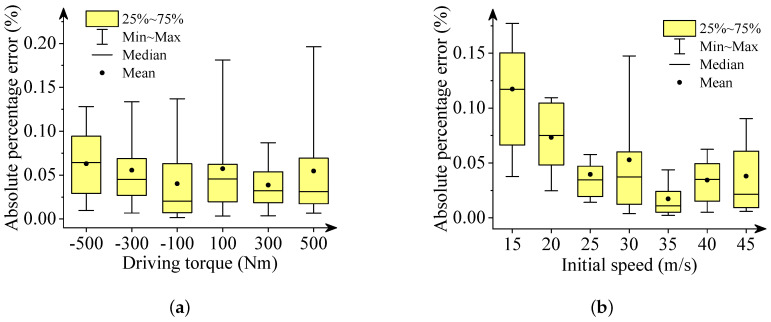
Box plots of absolute percentage error: final longitudinal distance. (**a**) Driving torque; (**b**) Initial speed.

**Figure 7:**
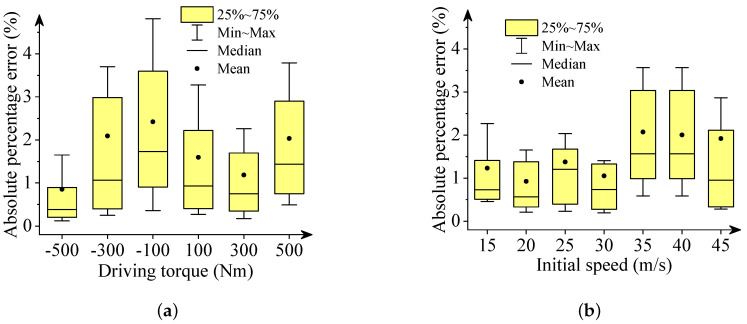
Box plots of percentage absolute error: final lateral distance. (**a**) Driving torque; (**b**) Initial speed.

**Figure 8:**
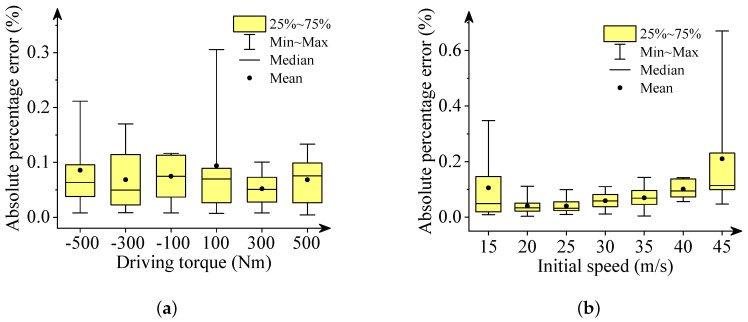
Box plots of absolute percentage error: final longitudinal velocities. (**a**) Driving torque; (**b**) Initial speed.

**Figure 9:**
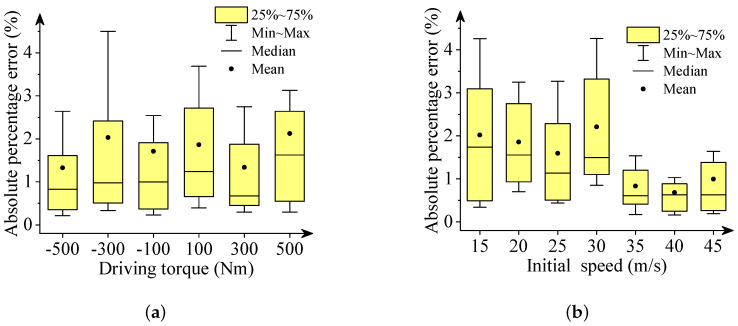
Box plots of absolute percentage error: final lateral velocities. (**a**) Driving torque; (**b**) Initial speed.

**Figure 10:**
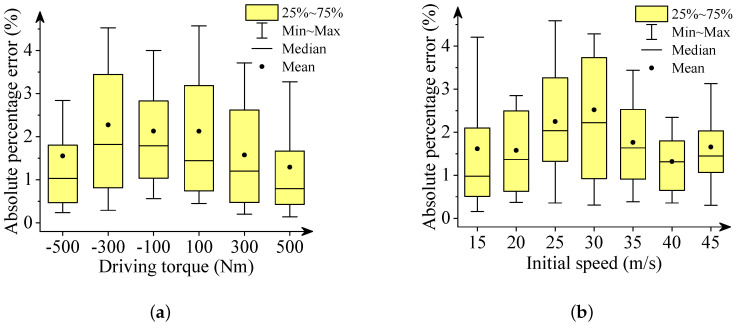
Box plots of absolute percentage error: yaw angle. (**a**) Driving torque; (**b**) Initial speed.

We used box plots, described in Figure 6, Figure 7, Figure 8, Figure 9 and Figure 10, to show the absolute percentage error between the results of the multibody model and the DNN model. The elements in the box plots generally include median, mean, upper quartile, lower quartile, upper limit, and lower limit. They can be used to clearly depict the data dispersion and bias of the dataset. They are not affected by outliers and can describe the discrete distribution of data in a relatively stable way. Different initial speeds and driving torques in a wide range were used to imitate various driving situations.

Two conclusions can be drawn based on the absolute percentage error analysis and results comparison.

- The DNN results of the longitudinal distance and speed fit the multibody model results well. The median, mean, and maximum absolute percentage errors are less than 1%.
- The lateral distance and speed, and yaw angle predicted by the DNN model fit the reference results (multibody model results) well. The median, mean, and maximum absolute percentage errors are less than 3%.

### 3.2. Model Accuracy and Efficiency

In this section, the accuracy of the DNN model was evaluated quantifiably by error functions. We also investigated its computational efficiency. The accuracy evaluation metrics include the mean absolute error (MAE), the mean absolute percentage error (MAPE), the maximum absolute error (ME), the root mean square error (RMSE), and $R^{2}$. $R^{2}$ represents the coefficient of determination. It is a statistical indicator that can be used to reflect the regression model to illustrate the reliability of the variable change. These error functions are described as:

(23) $$\begin{array}{cc} \; & \mathrm{ME}=\max_{1\leq i\leq n}\left\{\left|y_{i}-{\hat{y}}_{i}\right|\right\} \end{array}$$

(24) $$\begin{array}{cc} \; & \mathrm{MAE}=\frac{1}{n}\sum_{i=1}^{n}\left|y_{i}-{\hat{y}}_{i}\right| \end{array}$$

(25) $$\begin{array}{cc} \; & \mathrm{MAPE}=\frac{1}{n}\sum_{i=1}^{n}\left|\frac{y_{i}-{\hat{y}}_{i}}{y_{i}}\right| \end{array}$$

(26) $$\begin{array}{cc} \; & \mathrm{RMSE}=\sqrt{\frac{1}{n}{\sum_{i=1}^{n}\left(y_{i}-{\hat{y}}_{i}\right)}^{2}} \end{array}$$

(27) $$\begin{array}{cc} \; & R^{2}=1-\frac{{\overset{n}{\underset{i=1}{\sum }}\left(y_{i}-{\hat{y}}_{i}\right)}^{2}}{{\overset{n}{\underset{i=1}{\sum }}\left(y_{i}-{\bar{y}}_{i}\right)}^{2}} \end{array}$$

where, $y_{i}$ denotes the *i*-th value of the reference results, ${\hat{y}}_{i}$ denotes the *i*-th value of the DNN results, ${\bar{y}}_{i}$ denotes the *i*-th mean value of the DNN results, and *n* denotes the number of groups, which equals 7701 in this study. The five evaluation metrics were used to quantify model accuracy. The smaller the first four metrics are, the more accurate the DNN model is. However, for $R^{2}$, if its value is closer to 1, the DNN model fits well.

The results of accuracy analysis are described in Table 1. It can be see from Table 1 that the MAE, MAPE, ME, and RMSE of the vehicle states are relatively small, while $R^{2}$ is very close to 1. This indicates that the DNN model has high accuracy in predicting longitudinal-lateral dynamics of the vehicle. Furthermore, the prediction of longitudinal dynamics is slightly more accurate than the prediction of lateral dynamics.

To better visualize the high accuracy of the proposed DNN modelling method, the widely used backpropagation (BP) neural network and radical basis function (RBF) neural network were used as references for comparison. The solution accuracy was investigated in term of evaluation metrics, e.g., ME, MAE, MAPE, RSME, and $R^{2}$. The comparative results, taking yaw angle as an example, are described in Table 2. As presented in Table 2, the ME, MAE, MAPE, RSME of the DNN method are smaller than those of the BP and RBF methods, and $R^{2}$ of the DNN method is much closer to 1. It can be concluded that the DNN model is more accurate than the widely used BP and RBF models.

To investigate the computational efficiency of the DNN model, the CPU time of DNNs with different numbers of hidden layers were compared. The simulation times of the multibody model with different time-steps were measured for references. The results are shown in detail in Table 3. Note that the CPU time is the average time of ten random driving situations.

It is easy to observe in Table 3 that the CPU time for predicting longitudinal-lateral dynamics is approximately 1 ms with 2, 3, and 4 hidden layers. The computational efficiency is almost the same in these cases, which is very high and can be used for faster-than-real-time simulation. The CPU times of the multibody model to run a 5 s dynamic simulation are 3971 ms, 383 ms, and 38.1 ms, corresponding to the time-steps of 1 ms, 10 ms, and 100 ms, respectively. It can be seen that the DNN model is much faster than the multibody model for predicting the vehicle states.

Furthermore, to investigate the effects of dataset size on the accuracy of DNN model, different numbers of samples were used to train the DNN model. The MAPE was calculated to investigate how large the data size can predict the longitudinal-lateral dynamics effectively. It can be seen from Table 4 that the MAPE is large when 300 or 400 samples are used. It means that the DNN results are not very accurate. The maximum MAPE is about 2.5% when 500 samples are used to train the DNN model. The DNN model accuracy improves as the sample size increases. It can be concluded, for this vehicle example, that the DNN model accuracy is satisfy once the sample size reaches 500.

### 3.3. Model Verification and Discussion

**Figure 11:**
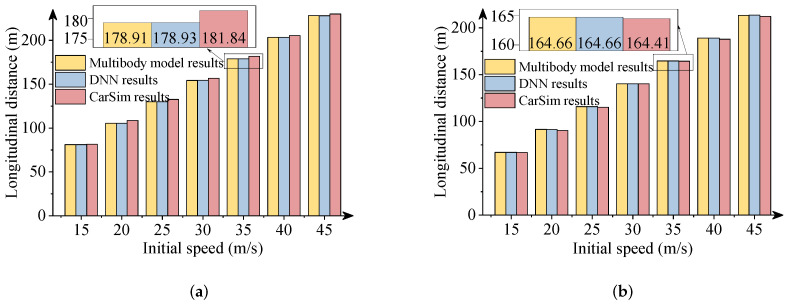
Comparison of longitudinal distances. (**a**) 300 Nm driving torque; (**b**) −300 Nm driving torque.

**Figure 12:**
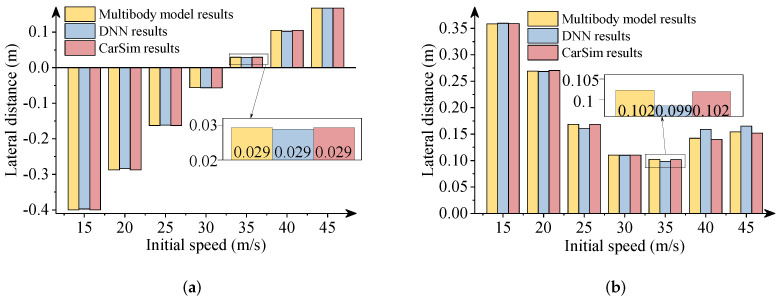
Comparison of lateral distances. (**a**) 300 Nm driving torque; (**b**) −300 Nm driving torque.

**Figure 13:**
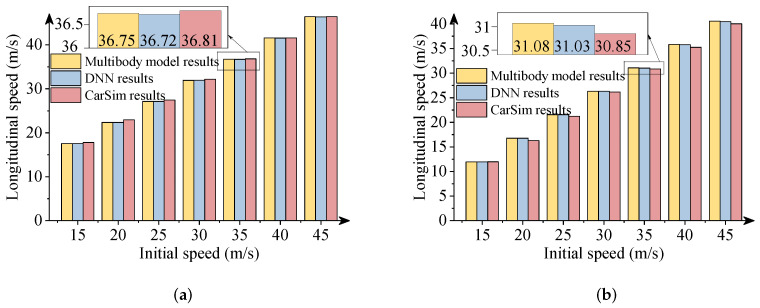
Comparison of longitudinal velocities. (**a**) 300 Nm driving torque; (**b**) −300 Nm driving torque.

In this section, the results obtained from the commercial software package CarSim were used to verify the effectiveness of the DNN model. It is noteworthy that vehicle simulations with the same parameters were performed in CarSim. Figure 11, Figure 12 and Figure 13 describe the comparative results when the −300 Nm and 300 Nm driving torques are imposed. The detailed views in these figures show the maximum differences between the DNN results and the CarSim results.

As presented in above figures, the predicted results via DNNs coincide with the numerical results of the software package CarSim. The DNN results also are consistent with the multibody model results. The driving situations imitate the vehicle’s decelerations and accelerations during a double-lane change maneuver with a wide range of initial speeds. As a result, the effectiveness of the DNN model to predict the vehicle’s longitudinal-lateral dynamics was verified. The DNN model can be trained offline according to a large quantity of vehicle data and used for the preview control in vehicles to improve the handling, performance, and road safety. This DNN model is particularly very useful for the preview control in autonomous vehicles for enhanced transportation safety. Note that the DNN model was developed for a specific vehicle system. Useful DNN models of different vehicle systems can be developed based on a similar modelling procedure.

## 4. Conclusions

In this study, a DNN model to predict the longitudinal-lateral dynamics of an autonomous vehicle was developed. A semirecursive multibody formulation was used to capture the vehicle characteristics. An algorithm to obtain the data of longitudinal-lateral dynamics and a DNN modelling approach were presented. It is significant that massive parallel computations can be performed by using GPUs that are especially suitable for the large volumes of data in autonomous vehicles. To verify the DNN model, the testing data were randomly selected for accuracy evaluation. Error functions also were used to quantifiably analyze the model accuracy. The computational efficiency and accuracy were investigated for neural networks with different structures. Finally, the results from a commercial software package CarSim were used to validate the proposed DNN model. The results indicated that the DNN model can predict the vehicle’s lateral-longitudinal dynamics accurately and efficiently. The DNN model used for modelling lateral, longitudinal, and vertical coupling dynamics will be investigated in the future. Overall, a DNN-based methodology was presented to predict the vehicle’s lateral-longitudinal dynamics in real time. It is suitable for the preview control of autonomous vehicles in complex environment to enhance the transportation safety.

## Figures and Tables

**Table 1:** The accuracy of the DNN model.

Vehicle Responses	ME (m, m/s, rad)	MAE (m, m/s, rad)	MAPE (%)	RMSE (m, m/s, rad)	$R^{2}$
Longitudinal distance	0.6927	0.0628	0.0498	0.0799	0.9999
Lateral distance	0.1348	0.0019	2.0064	0.0039	0.9995
Longitudinal speed	0.3463	0.0218	0.0682	0.0317	0.9999
Lateral speed	0.0489	0.0004	2.1642	0.0012	0.9984
Yaw angle	0.0012	0.0001	2.5313	0.0001	0.9989

**Table 2:** The accuracy of different neural network methods (yaw angle).

Neural Network Methods	ME (rad)	MAE (rad)	MAPE (%)	RMSE (rad)	$R^{2}$
RBF	0.0084	0.0115	188.50	0.0015	0.1660
BP	0.0019	0.0002	3.58	0.0001	0.9961
DNN	0.0012	0.0001	2.53	0.0001	0.9989

**Table 3:** CPU time of DNN and multibody models with different structures for 5 s dynamic simulation.

CPU Time (ms)	DNN Model with Different Hidden Layers			Multibody Model with Different Time-Steps		
	4	3	2	1 ms	10 ms	100 ms
Longitudinal distance	1.006	0.998	1.006	3971	383	38.1
Lateral distance	1.003	1.003	0.996			
Longitudinal speed	1.005	0.996	1.003			
Lateral speed	1.009	0.992	1.000			
Yaw angle	0.994	1.004	1.007			

**Table 4:** Comparison of different sample size (MAPE).

MAPE	The Number of Data Samples		
	300	400	500
Longitudinal distance	0.0640%	0.0399%	0.0498%
Lateral distance	14.9877%	6.8444%	2.0064%
Longitudinal speed	0.0829%	0.0514%	0.0682%
Lateral speed	4.8504%	4.3236%	2.1642%
Yaw angle	6.3860%	4.0779%	2.5313%

## Data Availability

Not applicable.

## References

[1] Severino A., Pappalardo G., Curto S., Trubia S., Olayode I.O. (2021). Safety Evaluation of Flower Roundabout Considering Autonomous Vehicles Operation. Sustainability.

[2] Cakici Z. (2021). Performance Evaluation of a Hybrid PSO Enhanced ANFIS Model in Prediction of Traffic Flow of Vehicles on Freeways: Traffic Data Evidence from South Africa. Infrastructures.

[3] Karlaftis M., Vlahogianni E. (2011). Statistical methods versus neural networks in transportation research: Differences, similarities and some insights. Transp. Res. Part C Emerg. Technol..

[4] Yim Y.U., Oh S.Y. (2004). Modeling of vehicle dynamics from real vehicle measurements using a neural network with two-stage hybrid learning for accurate long-term prediction. IEEE Trans. Veh. Technol..

[5] Pan C.Z., Lai X.Z., Yang S.X., Wu M. (2013). An efficient neural network approach to tracking control of an autonomous surface vehicle with unknown dynamics. Expert Syst. Appl..

[6] Da Lio M., Bortoluzzi D., Rosati Papini G.P. (2020). Modelling longitudinal vehicle dynamics with neural networks. Veh. Syst. Dyn..

[7] Fayyaz M., González-González E., Nogués S. (2022). Autonomous Mobility: A Potential Opportunity to Reclaim Public Spaces for People. Sustainability.

[8] Silva D.S., Csiszar C., Fldes D. (2021). Autonomous Vehicles and Urban Space Management. Sci. J. Silesian Univ. Technol. Ser. Transp..

[9] He Y., Csiszár C. (2021). Model for Crowdsourced Parcel Delivery Embedded into Mobility as a Service Based on Autonomous Electric Vehicles. Energies.

[10] Bartuska L., Labudzki R. (2020). Research of basic issues of autonomous mobility. Transp. Res. Procedia.

[11] Dudziak A., Stoma M., Kuranc A., Caban J. (2021). Assessment of Social Acceptance for Autonomous Vehicles in Southeastern Poland. Energies.

[12] Cai P., Mei X., Tai L., Sun Y., Liu M. (2020). High-Speed Autonomous Drifting with Deep Reinforcement Learning. IEEE Robot. Autom. Lett..

[13] Kuutti S., Bowden R., Jin Y., Barber P., Fallah S. (2020). A survey of deep learning applications to autonomous vehicle control. IEEE Trans. Intell. Transp. Syst..

[14] Horla D., Hamandi M., Giernacki W., Franchi A. (2021). Optimal Tuning of the Lateral-Dynamics Parameters for Aerial Vehicles with Bounded Lateral Force. IEEE Robot. Autom. Lett..

[15] Kumarawadu S., Lee T.T. (2006). Neuroadaptive combined lateral and longitudinal control of highway vehicles using RBF networks. IEEE Trans. Intell. Transp. Syst..

[16] Melzi S., Sabbioni E. (2011). On the vehicle sideslip angle estimation through neural networks: Numerical and experimental results. Mech. Syst. Signal Process..

[17] Ji X., He X., Lv C., Liu Y., Wu J. (2018). Adaptive-neural-network-based robust lateral motion control for autonomous vehicle at driving limits. Control Eng. Pract..

[18] Devineau G., Polack P., Altché F., Moutarde F. Coupled longitudinal and lateral control of a vehicle using deep learning. Proceedings of the 2018 21st International Conference on Intelligent Transportation Systems (ITSC).

[19] Acosta M., Kanarachos S. (2018). Teaching a vehicle to autonomously drift: A data-based approach using neural networks. Knowl.-Based Syst..

[20] Taghavifar H., Hu C., Taghavifar L., Qin Y., Na J., Wei C. (2020). Optimal robust control of vehicle lateral stability using damped least-square backpropagation training of neural networks. Neurocomputing.

[21] Kim D., Min K., Kim H., Huh K. (2020). Vehicle sideslip angle estimation using deep ensemble-based adaptive Kalman filter. Mech. Syst. Signal Process..

[22] Tork N., Amirkhani A., Shokouhi S.B. (2021). An adaptive modified neural lateral-longitudinal control system for path following of autonomous vehicles. Eng. Sci. Technol. Int. J..

[23] Šabanovič E., Žuraulis V., Prentkovskis O., Skrickij V. (2020). Identification of Road-Surface Type Using Deep Neural Networks for Friction Coefficient Estimation. Sensors.

[24] Zadeh A.G., Fahim A., El-Gindy M. (1997). Neural network and fuzzy logic applications to vehicle systems: Literature survey. Int. J. Veh. Des..

[25] Rosenblum M., Davis L.S. (1996). An improved radial basis function network for visual autonomous road following. IEEE Trans. Neural Netw..

[26] Kalkkuhl J., Hunt K.J., Fritz H. (1999). FEM-based neural-network approach to nonlinear modeling with application to longitudinal vehicle dynamics control. IEEE Trans. Neural Netw..

[27] Attia R., Orjuela R., Basset M. (2014). Combined longitudinal and lateral control for automated vehicle guidance. Veh. Syst. Dyn..

[28] Nguyen A.T., Dinh T.Q., Guerra T.M., Pan J. (2021). Takagi-Sugeno Fuzzy Unknown Input Observers to Estimate Nonlinear Dynamics of Autonomous Ground Vehicles: Theory and Real-Time Verification. IEEE/ASME Trans. Mechatron..

[29] Karkee M., Steward B.L., Kelkar A.G., Kemp Z.T. (2011). Modeling and real-time simulation architectures for virtual prototyping of off-road vehicles. Virtual Real..

[30] Bruni S., Meijaard J., Rill G., Schwab A. (2020). State-of-the-art and challenges of railway and road vehicle dynamics with multibody dynamics approaches. Multibody Syst. Dyn..

[31] García de Jalón J., Callejo A., Hidalgo A.F. (2012). Efficient Solution of Maggi’s Equations. J. Comput. Nonlinear Dyn..

[32] Pan Y., Callejo A., Bueno J.L., Wehage R.A., García de Jalón J. (2017). Efficient and accurate modeling of rigid rods. Multibody Syst. Dyn..

[33] Rodríguez J.I., Jiménez J.M., Funes F.J., García de Jalón J. (2004). Recursive and Residual Algorithms for the Efficient Numerical Integration of Multi-Body Systems. Multibody Syst. Dyn..

[34] García de Jalón J., Álvarez E., de Ribera F., Rodríguez I., Funes F., Ambrósio J. (2005). A Fast and Simple Semi-Recursive Formulation for Multi-Rigid-Body Systems. Advances in Computational Multibody Systems.

[35] Pan Y., Dai W., Xiong Y., Xiang S., Mikkola A. (2020). Tree-topology-oriented modeling for the real-time simulation of sedan vehicle dynamics using independent coordinates and the rod-removal technique. Mech. Mach. Theory.

[36] Pan Y., Xiang S., He Y., Zhao J., Mikkola A. (2020). The validation of a semi-recursive vehicle dynamics model for a real-time simulation. Mech. Mach. Theory.

[37] Liu Y., Yang D., Zhang C. (2018). Relaxed conditions for convergence analysis of online back-propagation algorithm with L2 regularizer for Sigma-Pi-Sigma neural network. Neurocomputing.

[38] Zou F., Shen L., Jie Z., Zhang W., Liu W. A sufficient condition for convergences of adam and rmsprop. Proceedings of the IEEE/CVF Conference on Computer Vision and Pattern Recognition.

[39] Pacejka H.B. (2012). Tire and Vehicle Dynamics.

[40] Xue Y., Wang Y., Liang J. (2022). A self-adaptive gradient descent search algorithm for fully-connected neural networks. Neurocomputing.

[41] Sun T., Li D. (2022). Sign Stochastic Gradient Descents without bounded gradient assumption for the finite sum minimization. Neural Netw..

